# Ferroelectric field effect transistors based on two-dimensional CuInP_2_S_6_ (CIPS) and graphene heterostructures

**DOI:** 10.1557/s43581-024-00109-y

**Published:** 2024-08-21

**Authors:** Maheera Abdul Ghani, Soumya Sarkar, Yang Li, Ye Wang, Kenji Watanabe, Takashi Taniguchi, Yan Wang, Manish Chhowalla

**Affiliations:** 1 Department of Materials Science and Metallurgy, University of Cambridge, 27 Charles Babbage Road, Cambridge, CB3 0FS UK; 2 Research Center for Electronic and Optical Materials, National Institute for Materials Science, 1-1 Namiki, Tsukuba, Ibaraki 305-0044 Japan; 3 Research Center for Materials Nanoarchitectonics, National Institute for Materials Science, 1-1 Namiki, Tsukuba, Ibaraki 305-0044 Japan

**Keywords:** van der Waals, graphene, ferroelectricity, 2D materials, nanoelectronics

## Abstract

**Abstract:**

Heterostructures of two-dimensional (2D) materials comprise clean van der Waals (vdW) interfaces that can facilitate charge or energy transfer. Recently, the 2D ferroelectric CuInP_2_S_6_ (CIPS) has been integrated with graphene and other 2D materials to realize potentially novel low energy electronic devices. However, the influence of 2D CIPS on the properties of graphene and doping across the vdW interface has not been studied in detail. Here, we study graphene field effect transistors (FETs) with CIPS as the top gate. We find that CIPS leads to modulation of the graphene Fermi level due to local doping. We also find polarization-induced hysteresis in CIPS-gated graphene FETs. Electrical transport measurements from 50 to300 K show that above 200 K, the ferroelectric response decreases. As a result, the hysteresis voltage windows in the graphene ferroelectric FETs (FeFET) transfer curves decrease above 200 K. Our results show that interfacial remote doping affects the macroscopic polarization and performance of CIPS-based graphene FeFETs.

**Graphical abstract:**

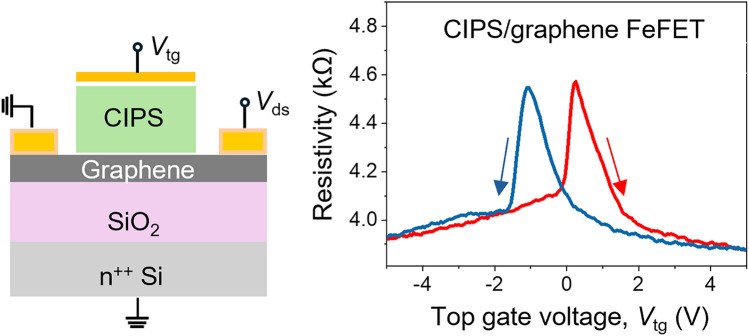

**Highlights:**

- This research studies the temperature-dependent local doping across a vdW ferroelectric/2D channel interface that affects the transport properties of ferroelectric field effect transistors (FeFETs).
- Experimental findings showed ferroelectric polarization switching-based hysteresis in CuInP_2_S_6_-gated graphene FeFETs.

**Discussion:**

- vdW ferroelectrics that can be scaled to atomic layer thicknesses are useful for miniaturised low energy electronics.
- Understanding the interface charge or energy transfer in vdW ferroelectrics is essential for their integration into current or future technologies.

**Supplementary Information:**

The online version contains supplementary material available at 10.1557/s43581-024-00109-y.

## Introduction

In bulk ferroelectric materials, inversion symmetry breaking gives rise to electrically switchable polarization, that originates from spontaneous ordering of electrical dipoles.^1^ Electrical dipoles in a ferroelectric are strongly coupled with the charge and lattice degrees of freedom.^2^ Consequently, the polarization is affected by strain, defects, and doping.^3–5^ These effects are pronounced at the interfaces, and they affect the properties of devices based on ultrathin ferroelectrics.^6,7^ The role of interface effects on the performance of conventional oxide-based ferroelectrics has been widely investigated.^3^ Compared to conventional oxide-based ferroelectrics which possess a rigid lattice structure defined by ionic bonding, van der Waals (vdW) ferroelectrics (such as CuInP_2_S_6_ (CIPS), α-In_2_Se_3_) exhibit a layered structure with weak interlayer bonding.^8–12^ As a result, interfaces of vdW ferroelectrics are electronically and atomically sharp, devoid of dangling bonds making it easy to form heterostructures that could be compatible with industrial processes.^11,13^ Furthermore, owing to their low intrinsic polarization, vdW ferroelectrics exhibit immunity to depolarization fields.^14^ These properties permit vdW ferroelectrics to be scaled to atomic layer thicknesses, useful for miniaturized low energy nanoelectronics. The bandgap of vdW ferroelectrics is lower than traditional oxide ferroelectrics which is of significance for photovoltaics and photonic devices.^15,16^ However, the low bandgap semiconducting nature exacerbates interface effects related to defects and charge transfer. These interface effects lead to inconsistent characteristics of devices based on vdW ferroelectrics, such as asymmetric hysteresis related to defects and traps.^17^ It is therefore important to identify the influence of interface effects on the polarisation of vdW ferroelectrics.

It is challenging to probe interface effects with metal electrodes due to their high carrier density, which leads to extremely short Fermi screening lengths (< 1 Å).^18^ On the other hand, graphene electrodes due to their unique band structure allow electrostatic tunability of Fermi level by approximately 1 eV and have long screening length of nearly 1 nm.^19^ In this work, we use a graphene field effect transistor (FET) to monitor interface effects on the ferroelectricity of vdW ferroelectric CIPS.

CIPS is a transition metal thiophosphate that exhibits several intriguing properties. These include the coexistence of out-of-plane ferroelectricity and ionic conductivity at room temperature, negative piezoelectricity, and bulk photovoltaic effect.^16^ The ferroelectric polarization in CIPS is attributed to the displacement of the Cu^+^ ion within the sulphur octahedra.^20^ Further, at elevated temperatures and larger electric fields, the increased displacement of Cu^+^ ions screens the ferroelectric polarization.^21^ Here, we describe how electrical transport through graphene is modulated by placing CIPS as a top gate dielectric layer. We find that placing CIPS on top of graphene locally dopes graphene with electrons. The doping is temperature-dependent and related to the displacement of Cu^+^ ion within the CIPS plane at higher temperatures (*T* > 200 K).^20^ We demonstrate CIPS-gated graphene ferroelectric FETs (FeFETs) that exhibit hysteresis due to polarization switching. Through temperature-dependent electrical characterization, we show how the properties of graphene FET are affected by doping from CIPS.

## Results and discussions

**Figure 1:**
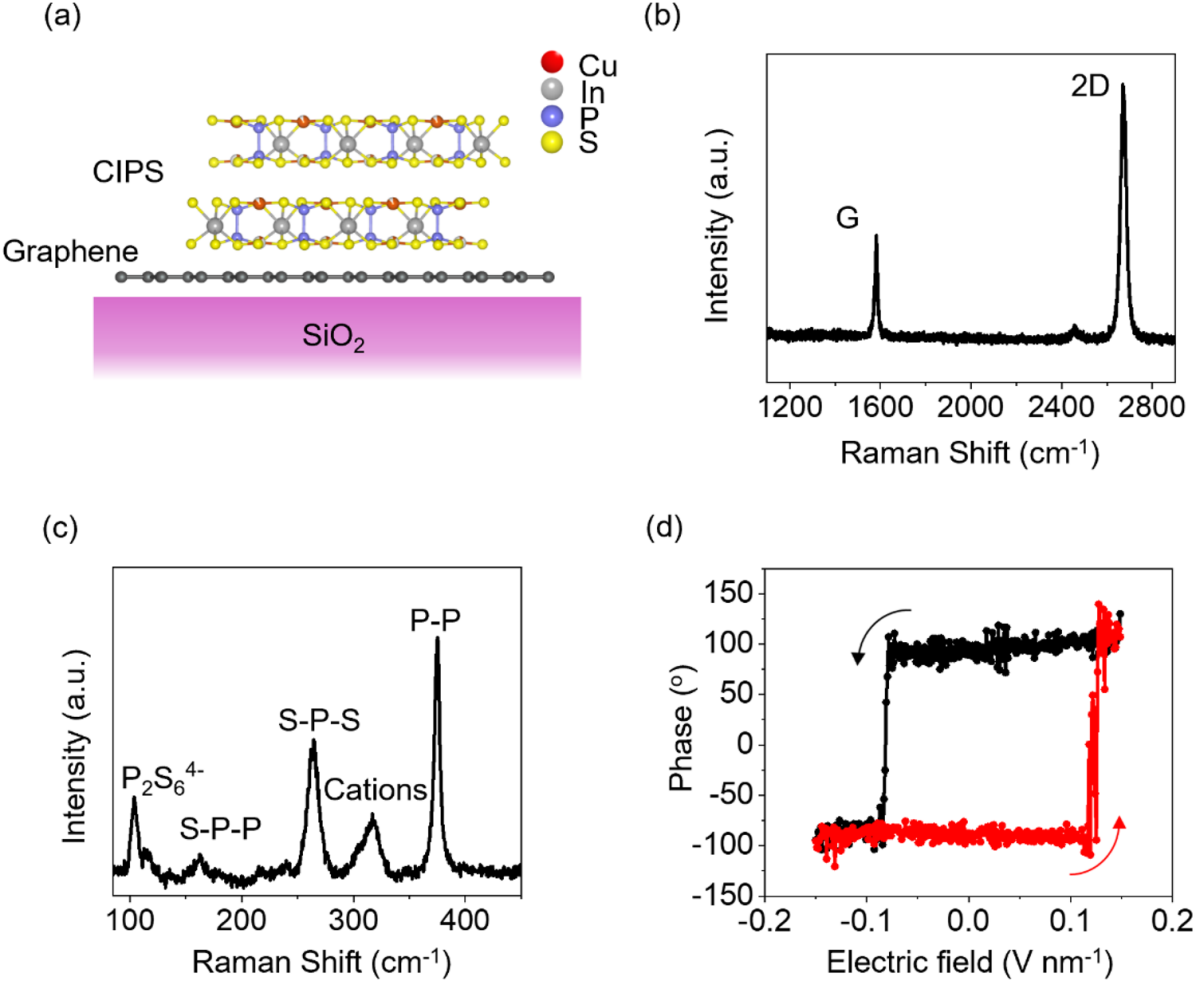
(a) Schematic of CIPS/graphene vdW heterostructure on a SiO_2_ substrate. Raman spectra for (b) monolayer graphene/SiO_2_ and (c) 45-nm-thick CIPS/SiO_2_. (d) Out-of-plane phase measurements obtained by piezoresponse force microscopy (PFM) on a CIPS/Au/SiO_2_ test device. The red and black curves indicate direction of voltage sweep from negative to positive and negative to positive, respectively.

Schematic of CIPS/graphene vdW heterostructures on SiO_2_ studied here are shown in Fig. 1a. Monolayer graphene flakes were exfoliated from a high-quality bulk graphite crystal on a 285-nm-thick SiO_2_ substrate. The CIPS flakes were exfoliated from a bulk crystal and subsequently dry-transferred onto graphene. The thickness of the CIPS in Fig. 1 was found to be 45 nm from atomic force microscopy (AFM) [see Figure S1 of supporting information (SI)].

Figure 1b shows the Raman spectrum of monolayer graphene on SiO_2_ showing the characteristic G peak at 1584 cm^−1^ and 2D peak at 2674 cm^−1^.^22^ Figure 1c shows the Raman spectrum of CIPS on SiO_2_. The peak at 101 cm^−1^ is related to the anionic (P_2_S_6_^4–^) vibrations, and the peaks at 161 cm^−1^, 263 cm^−1^, and 374 cm^−1^ correspond to the S-P-P, S-P-S, and P-P modes, respectively.^23^ The peak at 316 cm^−1^ is related to the cationic (In^3+^ and Cu^+^) vibrations and is a signature of the ferroelectric phase.^23^ The ferroelectric properties of CIPS were characterized by piezoresponse force microscopy (PFM). Figure 1d shows the out-of-plane phase PFM measurements on 45-nm-thick CIPS/Au/SiO_2_ test device. The observed hysteresis loop is a signature of ferroelectricity.^8^ Furthermore, the PFM amplitude and phase images of the CIPS flake were mapped to show the presence of polar domains (see Sect. 2 in SI).

**Figure 2:**
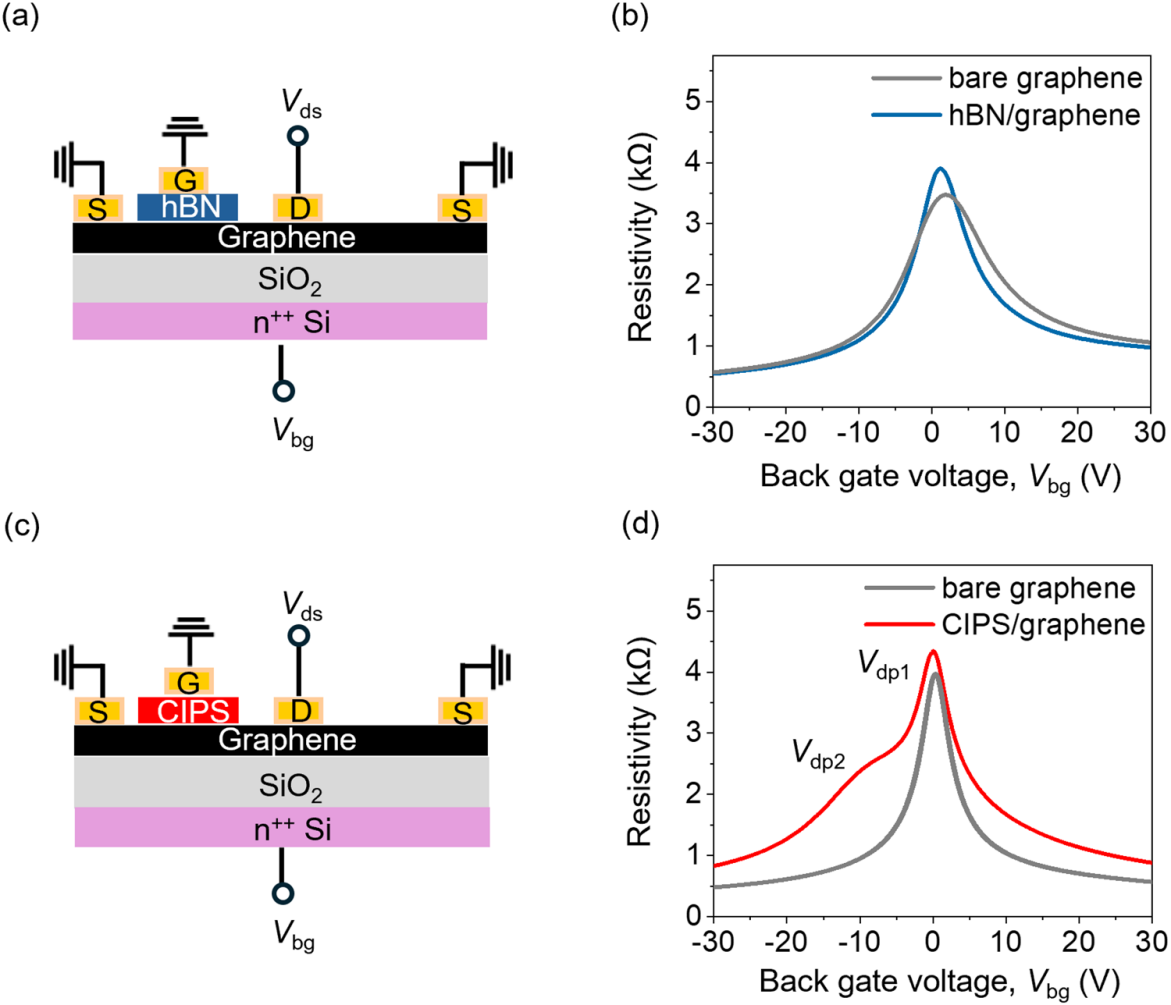
(a) Schematic of two graphene FETs fabricated on the same monolayer graphene flake. One of the FETs is covered with a 49-nm-thick hBN flake. (b) Resistivity of graphene measured as a function of back gate voltage, *V*_bg_ for bare graphene channel (grey curve) and graphene covered with hBN (blue curve). (c) Schematic of two graphene FETs fabricated on the same monolayer graphene flake. One of the FETs is covered with a 45-nm-thick CIPS flake. (d) Resistivity of graphene measured as a function of back gate voltage, *V*_bg_ for bare graphene channel (grey curve) and graphene covered with CIPS (red curve). All measurements are performed at room temperature. In both back gated FET measurements, the top dielectric (CIPS and hBN) was connected to the ground. In device schematic shown in (a) and (c), S is source electrode, D is drain electrode, G is top gate electrode, *V*_ds_ is drain source voltage, and *V*_bg_ is back gate voltage.

Next, we probe the electrical properties of the CIPS/graphene interface. For this, we compare the electrical characteristics of a CIPS-covered graphene FET with a bare graphene FET and hBN-covered graphene FET. CIPS-covered graphene and bare graphene FETs were fabricated on same flake to reduce the impact of device-to-device variations. Similar process was followed for hBN-covered graphene FET. For this study, we have chosen hBN-covered graphene FET as a reference because hBN is a wide bandgap insulator with an atomically sharp interface.^24^ The hBN flake was exfoliated from a high-quality crystal grown via high pressure and high temperature (HP–HT) method which is known to have a low defect density.^25^ Figure 2a shows the device schematic of two monolayer graphene FETs on a Si^++^/SiO_2_ substrate that were fabricated on the same graphene flake. One of the FETs was covered with a 49-nm-thick hBN layer (see corresponding AFM height profile in Figure S3). The resistivity as a function of back gate voltage at room temperature is presented in Fig. 2b. The transfer curves were obtained by forward and backward sweeps of the back gate voltage. The fact that no hysteresis is observed in the transfer curves indicates that the carrier transport is not limited by interfacial trap states.^26,27^ The room-temperature field effect mobility for our graphene samples is estimated to be 4000 cm^2^ V^−1^ s^−1^.

**Figure 3:**
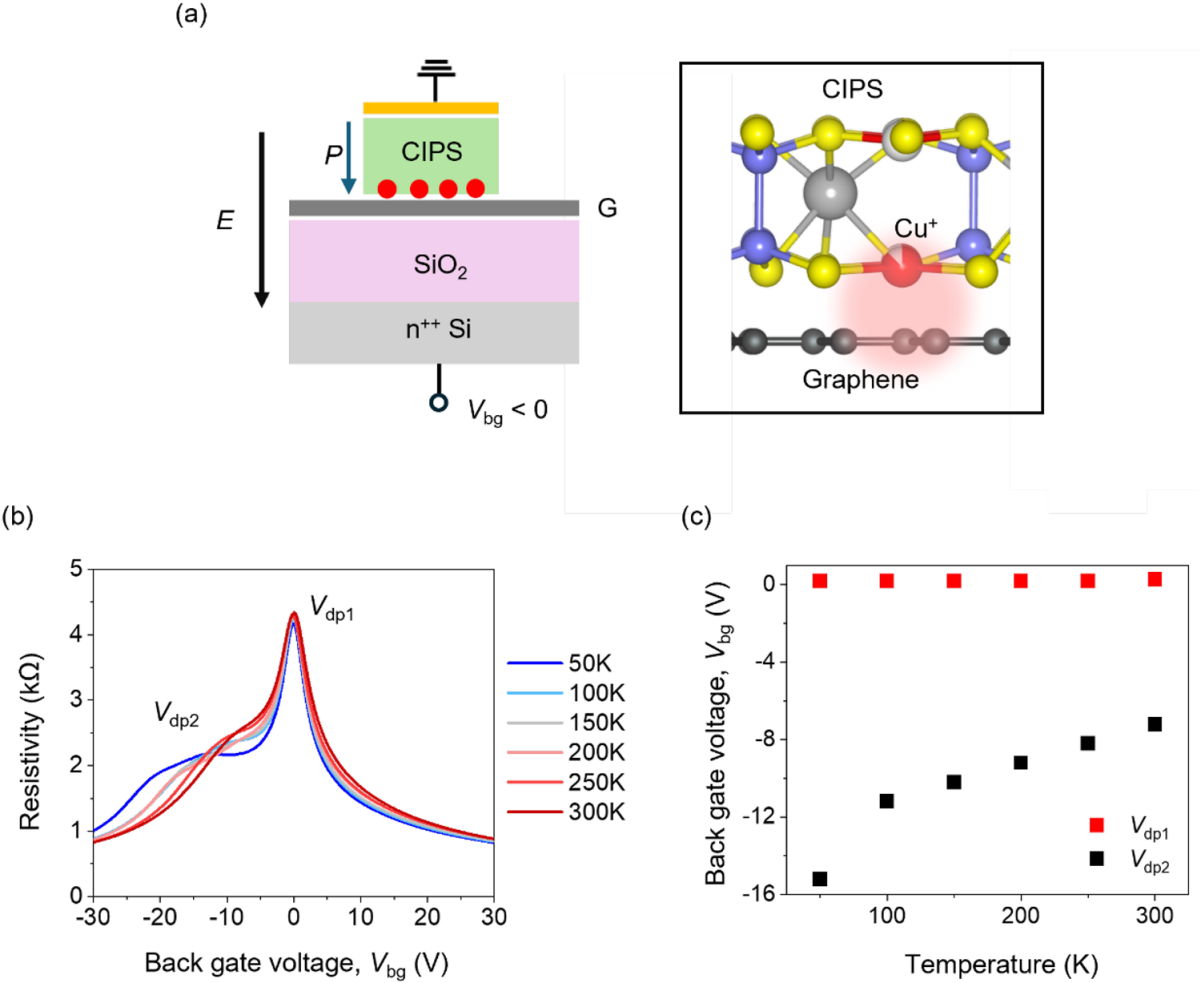
(a) Schematic illustration of alignment of Cu^+^ ions at the CIPS/graphene interface. *P* indicates polarization direction in CIPS, *E* is the electric field direction, *V*_bg_ is back gate voltage, and red circles represent Cu^+^ ion at the CIPS/graphene interface. (b) Temperature-dependent resistivity as a function of back gate voltage, *V*_bg_ for SiO_2_-gated graphene FET covered with CIPS. (c) Shift of *V*_dp2_ and *V*_dp1_ as a function of temperature.

To understand the effect of ferroelectric CIPS on the transport of graphene, we fabricate a similar device (see schematic in Fig. 2c) with two FETs on a single monolayer graphene flake—one bare and the other covered with CIPS. The optical microscope images of devices are shown in Sect. 4 of SI. The resistivity as a function of back gate voltage at room temperature for these two FETs is shown in Fig. 2d. We do not observe any hysteretic transfer characteristics, which again confirms that the carrier transport in the CIPS-covered graphene FET is not limited by interfacial trap states. Further, the linear output characteristics *(I*_ds_*–V*_ds_) at different back gate voltages (as shown in Figure S5 of SI) show ohmic nature of the In/Au vdW metal contacts.^28^ However, we observe an additional resistivity peak for the graphene FET covered with CIPS at back gate voltage of − 7.2 V along with primary Dirac peak (*V*_dp1_) positioned at back gate voltage of 0.3 V. The appearance of this second resistivity peak (*V*_dp2_) has been attributed to local modulation of the Fermi level due to local doping (Labelled in Fig. 2d).^29^ In our case, appearance of this *V*_dp2_ in the hole transport region (*V*_bg_ < 0) suggests localized n-doping.^29,30^ The localized n-doping should arise in the graphene channel that is covered by CIPS as the rest of the channel is p-doped by the back gate. The localized n-doping leads to formation of a local p–n junction in the graphene channel that is covered by CIPS. The origin of *V*_dp2_ and the local n-doping is likely related to the position of Cu^+ ^ions in the sulphur octahedral framework of CIPS.^31,32^ In response to an applied electric field, the Cu^+^ ions in CIPS can occupy a position either at the top or bottom of the sulphur octahedra, which represent the two stable ferroelectric polarization states (see Sect. 6 in SI).^33,34^ When *V*_bg_ < 0, the direction of applied vertical electric field points downwards towards the CIPS/graphene interface (as shown in Fig. 3a). This causes the polarization in CIPS to point downwards and the Cu^+^ ions occupy the bottom of the octahedra which leads to localized n-doping.

To further verify the origin of localized n-doping, we performed temperature-dependent transport measurements on CIPS/graphene FET (device schematic shown in Fig. 2c) as the Cu^+^ ion position in CIPS is known to vary with temperature.^20,33,35–37^ The temperature-dependent transfer characteristics from 50 to 300 K are shown in Fig. 3b. The measurements were performed at a safe voltage and temperature range to avoid conduction of Cu^+^ ions across the CIPS flake as observed in previous studies for high electric fields and temperatures.^33,38,39^ The position of the *V*_dp1_ is insensitive to changes in temperature, while the voltage at which we observe the *V*_dp2_ shifts towards *V*_dp1_ as the temperature is increased. This is manifested in the observed decrease in their separation (Δ*V)* from − 15.6 to − 7.2 V as shown in Fig. 3c. At low temperature (*T* = 50 K), the downward position of the Cu^+^ ion is spatially localized within the sulphur octahedra, and we observe local remote doping. As temperature increases, the Cu^+^ ions become relatively mobile hence the remote doping effect is delocalized. We have also measured temperature-dependent transfer characteristics of a bare graphene FET (shown in Figure S7 of SI) that does not show such any additional resistivity peaks.

**Figure 4:**
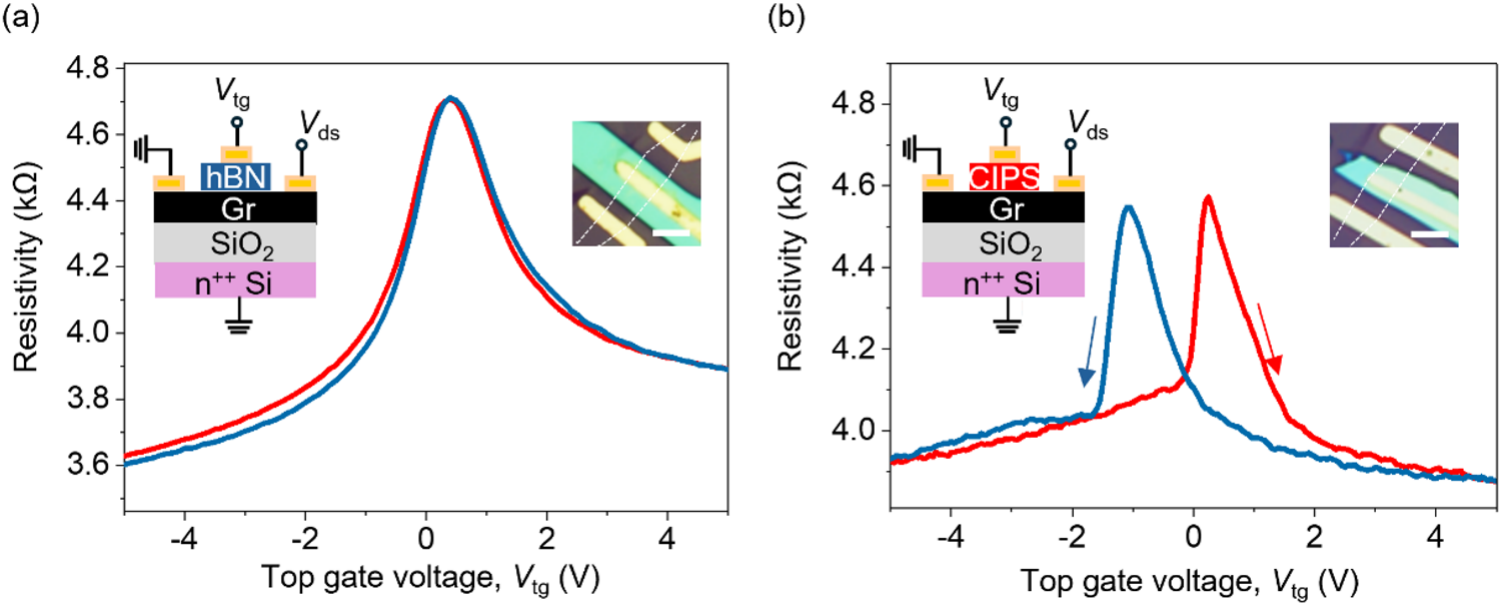
(a) Resistivity versus top gate voltage, *V*_tg_ applied across hBN for the hBN-gated graphene FET. The inset on the left shows the schematic of the device. The inset on the right shows an optical micrograph of the device. Scale bar is 5 µm. (b) Resistivity versus top gate voltage, *V*_tg_ applied across CIPS for the CIPS-gated graphene FET. The inset on the left shows the schematic of the device. The inset on the right shows an optical micrograph of the device. Scale bar 5 µm. The red and blue curves are forward and backward voltage sweeps, respectively. *V*_ds_ is drain source voltage and *V*_tg_ is top gate voltage.

Our results suggest that Cu^+^ ion displacement in CIPS can cause localized n-doping in graphene, we have investigated its effect on the electrical characteristics of a top-gated CIPS/graphene FeFET. To verify the ferroelectric effect, we first measure the electrical characteristics of a top-gated graphene FET with hBN as the gate dielectric (schematic of the device in the inset of Fig. 4a). The bottom Si gate was grounded for the top gate measurements. The resistivity versus top gate voltage on hBN is shown in Fig. 4a. The resistivity of the graphene channel is modulated, and a single Dirac peak is observed at a top gate voltage of 0.4 V for both forward and backward voltage sweeps. Next, we measure the electrical characteristics of top-gated graphene FET with CIPS as the gate dielectric (schematic of the device in the inset of Fig. 4b). In contrast to hBN, the forward and backward scans of the resistivity versus top gate voltage show hysteresis (Fig. 4b), exhibiting two maxima at + 0.28 V and − 1.1 V for Dirac peak position for forward sweep (*V*_Forward_) and backward sweep (*V*_Backward_), respectively. Further, the hysteresis of resistivity is clockwise for electrons (*V*_tg_ > 0) and anticlockwise for holes (*V*_tg_ < 0). These are clear signatures of polarization switching-related ferroelectric modulation of carriers in the graphene channel.^8^

**Figure 5:**
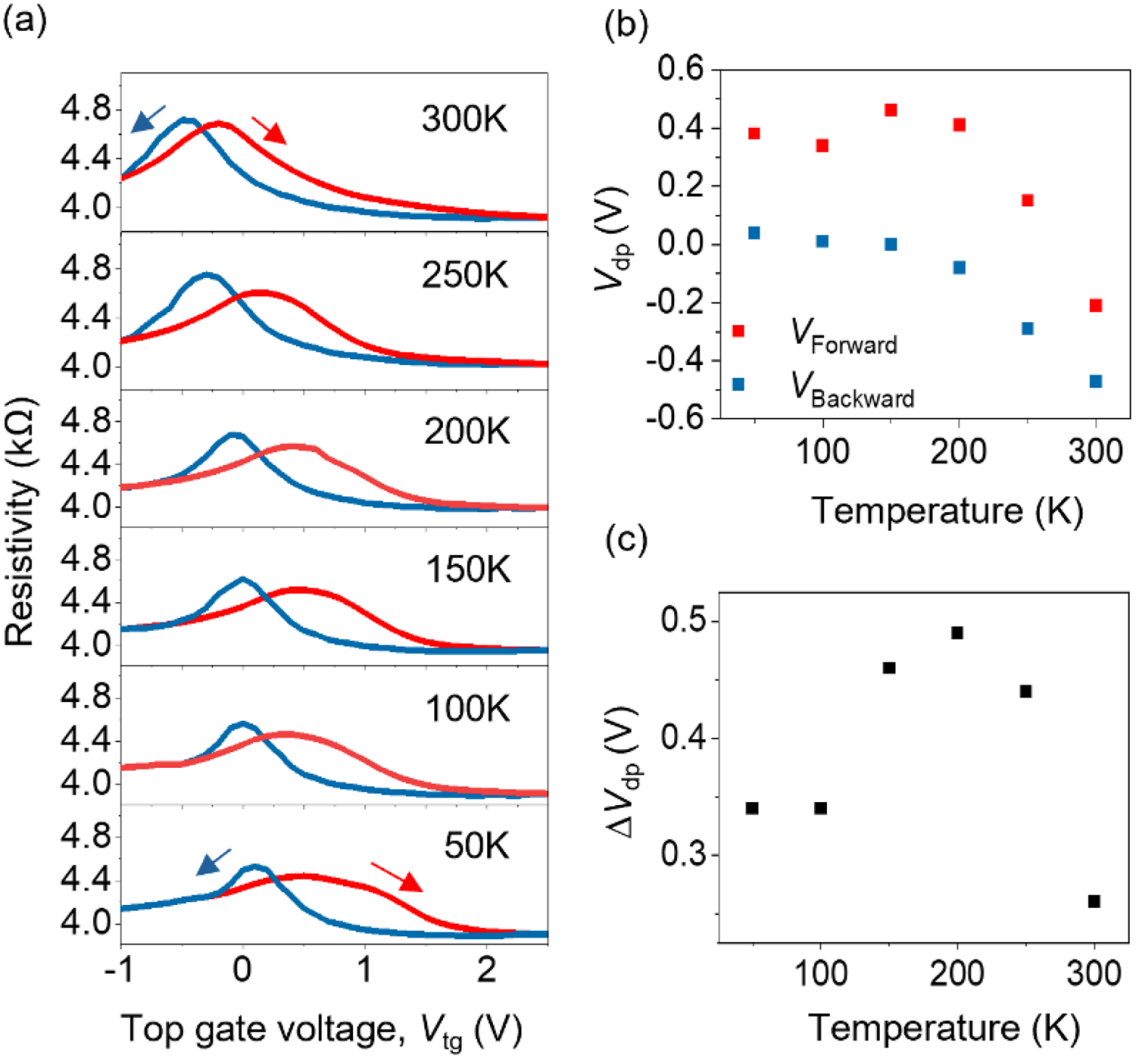
(a) The resistivity versus top gate voltage, *V*_tg_ hysteresis curves of a CIPS/graphene FeFET with temperature from 50 to 300 K. Temperature-dependent electrical transport measurements were investigated in the vacuum. The red and blue curves are forward and backward voltage sweeps, respectively. (b) Change in Dirac peak position for forward voltage sweep (*V*_forward_) and backward voltage sweep (*V*_Backward_) as a function of temperature. (c) Difference in *V*_Forward_ and *V*_Backward_, ∆*V*_dp_ with increasing temperature.

As shown earlier in Fig. 3, the localized n-doping from CIPS is temperature dependent. To correlate the temperature dependence of doping on electrical characteristics of the FeFETs, we measured resistivity as a function top gate voltage characteristics of a CIPS/graphene FeFET from 50 to 300 K (Fig. 5a). At 50 K, additional localized electron doping from the CIPS causes an asymmetry in the ferroelectric polarization for the two sweep directions, leading to asymmetric peaks in FeFET characteristics. We observed that the peak at the n-type region is wider compared to the p-type region. As the temperature increases, the peaks become more symmetric. This can be attributed to the presence of localized electron doping at lower temperatures which broadens the resistivity peak. We calculate the shift of resistivity peak positions, *V*_Forward_ and *V*_Backward_ as a function of the temperature in Fig. 5b. With the increase in temperature, *V*_Forward_ shifts from 0.39 to − 0.2 V and *V*_Backward_ shifts from 0.04 to − 0.47 V. Both *V*_Forward_ and *V*_Backward_ shift towards negative voltages that suggests with increase in temperature, the localized electrons become delocalized and contribute to the conductivity of the whole graphene channel. This results in n-doping of CIPS-based graphene FeFET. The voltage difference between the two Dirac peaks, *∆V*_dp_ (*V*_Forward_ and *V*_Backward_), is a signature of the induced ferroelectric polarization. As shown in Fig. 5c,* ∆V*_dp_ increases from 50 to 200 K. It has been previously shown that above 200 K, the Cu^+^ ion displacement increases and ionic conduction pathways are activated that screen the ferroelectric response. As a result, *∆V*_dp_ decreases above 200 K. ^20,38^

## Conclusion

We have demonstrated that a graphene FET is strongly influenced by dopants in vdW ferroelectric CIPS. The Cu^+^ ions cause n-doping in graphene. The dopants are spatially localized at low temperatures. Higher temperatures activate conduction pathways for the Cu^+^ ions causing the doping to delocalize. Finally, we show how doping across the ferroelectric graphene interface influences the properties of a CIPS-based FeFET. We demonstrate the influence of temperature-dependent polarization in CIPS on hysteresis of graphene FeFET transfer characteristics. Our results provide a better understanding of the properties of devices that are based on CIPS, ferroelectrics that are also ionic conductors, and other semiconducting vdW ferroelectrics. Research on CIPS-based FeFETs is still in early stages compared to HZO and AlScN FeFETs. However, CIPS has sharp interfaces which allow formation of functional heterostructures. FeFETs with a vdW interface between the channel and the gate dielectric are ideal to improve gate control and device performance.^40–42^ This will enable low energy electronics based on FeFETs using 2D materials.

## Experimental section

### CIPS and graphene sample preparation

CIPS and graphene samples were obtained by mechanical exfoliation^43^ from bulk crystals (2D Semiconductors). The flakes were exfoliated on 285-nm-thick SiO_2_ substrate using adhesive blue tape. Prior to this, the substrates were thoroughly cleaned for 15 min in acetone, isopropyl alcohol (IPA), and deionized water to remove any organic residues. The thicknesses and surface topography of flakes were confirmed by optical contrast, AFM, and Raman spectroscopy.

### AFM and PFM

AFM imaging was performed using a Bruker Icon system in ambient conditions using a scan rate of 0.6 Hz. The AFM images were analysed using Gwyddion 2.60 software package to determine height profile. Out-of-plane PFM measurements were carried out on Bruker Icon AFM system in the piezoresponse mode. PFM phase hysteresis loops were measured by recording the piezoresponse phase signals.

### Raman spectroscopy

Raman spectra were acquired using a Horiba LabRAM Evolution Raman microscope with 532 nm laser excitation and 1800 l/mm grating. The laser power was kept below 100mW to avoid damage to the flakes.

### Device fabrication

hBN and CIPS flakes were mechanically exfoliated onto PDMS stamp and stacked on mechanically exfoliated monolayer graphene using a dry transfer stage. We avoided the CIPS surface to come in contact with a polymer layer during the dry transfer process to reduce the effect of interfacial traps. The samples were spin-coated with MMA/PMMA resist for electron beam lithography (EBL). The EBL was carried out to pattern In/Au electrodes. Indium metal (8 V) was deposited in e-beam evaporator at a low rate of 0.1 Å/s, followed by a deposition of gold metal (70 nm) at 1 Å/s. The metal depositions were performed at a chamber pressure of < 10^−7^ torr.

### Electrical characterization

Electrical transport measurements were performed in the Lakeshore Cryogenic vacuum probe station with a Keithley 4200 semiconductor analyser under vacuum of < 10^−6^ torr and temperature of 50 K–300 K.

## Supplementary Information

Below is the link to the electronic supplementary material.

Supplementary file1 (PDF 2302 KB)
