# Supplementary material for: Ferroelectric field effect transistors based on two-dimensional CuInP2S6 (CIPS) and graphene heterostructures
Source: MRS Energy Sustain. 2024 Aug 21;11(2):616–23. doi: 10.1557/s43581-024-00109-y (PMC11564258; doi:10.1557/s43581-024-00109-y)
Supplement: Supplementary file 1 — Supplementary file1 (PDF 2302 KB) [file 43581_2024_109_MOESM1_ESM.pdf]

Supporting Information

Ferroelectric field effect transistors based on two dimensional $CuInP_2S_6$ (CIPS) and graphene heterostructures

Maheera Abdul Ghani[1], Soumya Sarkar[1], Yang Li[1], Ye Wang[1], Kenji Watanabe[2], Takashi Taniguchi[3], Yan Wang[1], Manish Chhowalla[1*]*

[1]Department of Materials Science and Metallurgy, University of Cambridge, 27 Charles Babbage Road, Cambridge CB3 0FS, United Kingdom

[2]Research Center for Electronic and Optical Materials, National Institute for Materials Science, 1-1 Namiki, Tsukuba, Ibaraki 305-0044, Japan

[3]Research Center for Materials Nanoarchitectonics, National Institute for Materials Science, 1-1 Namiki, Tsukuba, Ibaraki 305-0044, Japan

✉ Correspondence should be addressed to ss2806@cam.ac.uk, mc209@cam.ac.uk

Section 1: Atomic force microscopy (AFM) image of CIPS flake

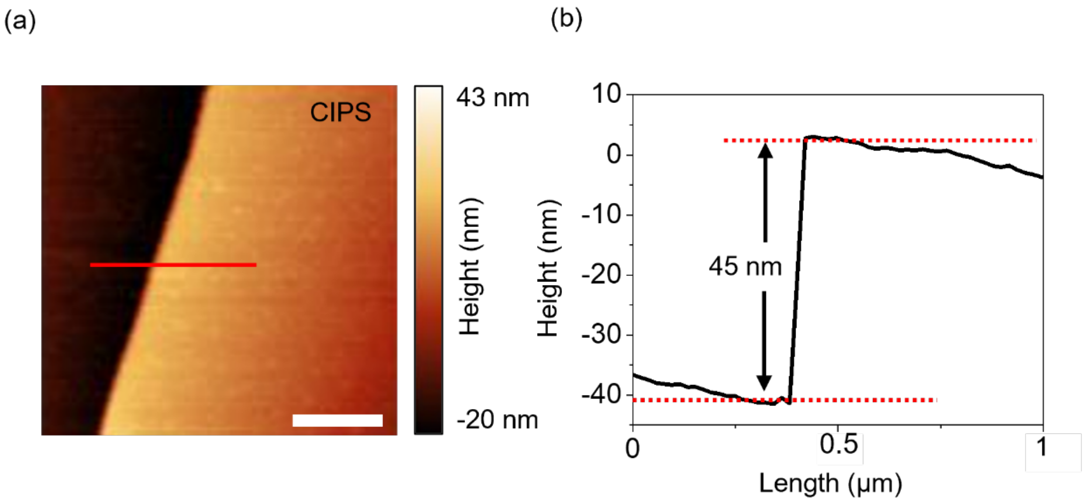

Figure S1. (a) AFM image (2×2 μm^2) and (b) corresponding AFM height profile of CIPS flake of line shown in (a). Scale bar in (a) is 500 nm.

Section 2: Piezoresponse force microscopy (PFM) images of CIPS flake

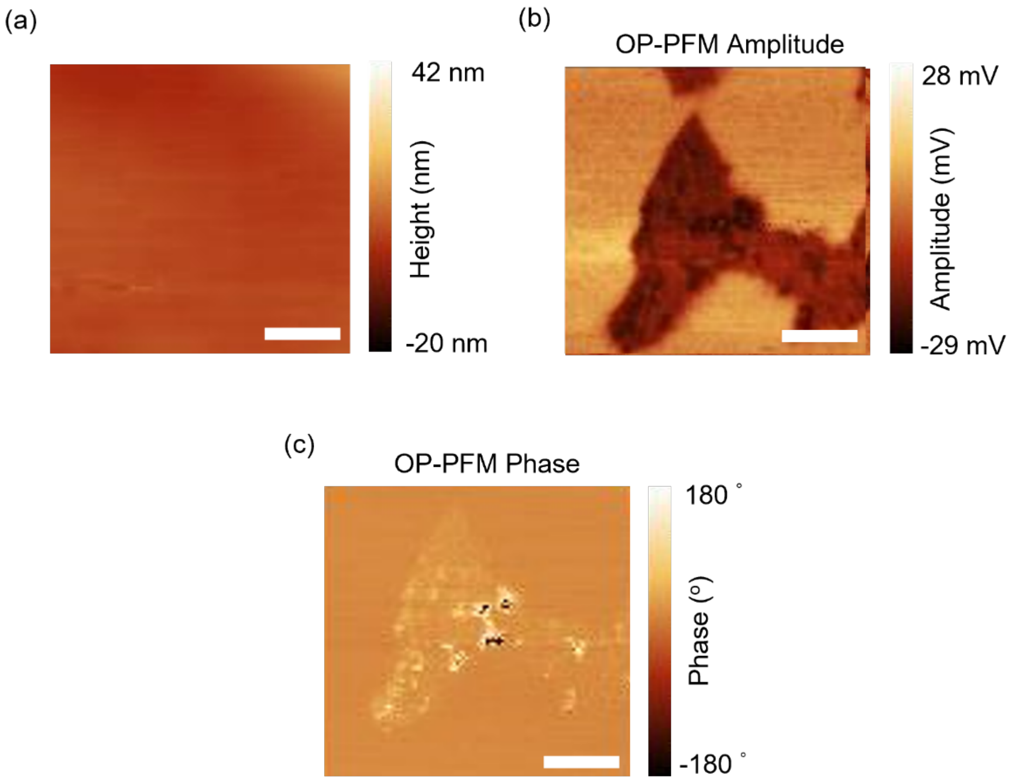

Figure S2. AFM and piezoresponse images of CIPS flake. (a) AFM topography (b) out of plane (OP) PFM amplitude and (c) OP PFM phase image of CIPS flake. Scale bar is 1 μm.

Section 3: Atomic force microscopy (AFM) image of hBN flake

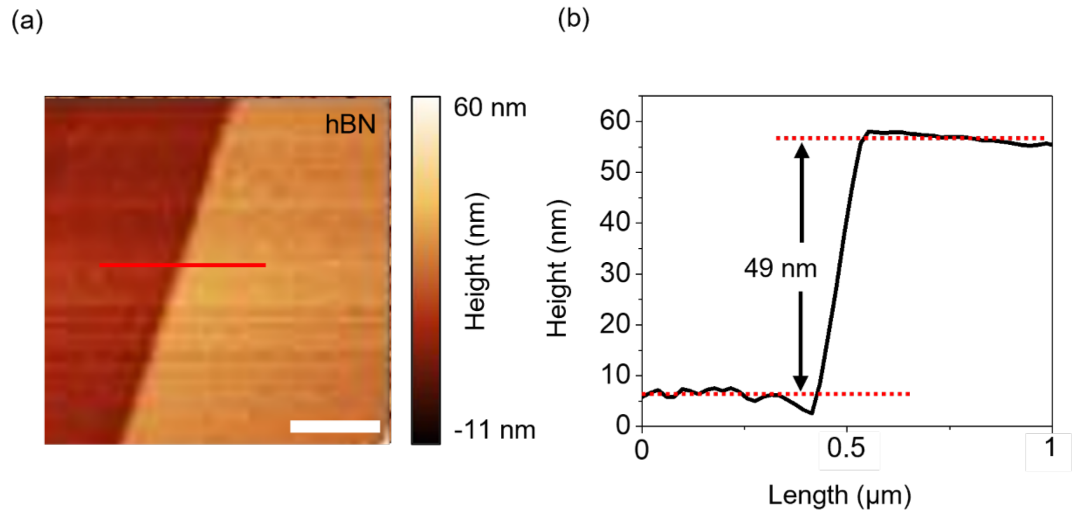

Figure S3. (a) AFM image (2×2 µm^2) and (b) corresponding AFM height profile of hBN flake of line shown in (a). Scale bar in (a) is 500 nm.

Section 4: Optical microscope images of hBN/graphene and CIPS/graphene FET device

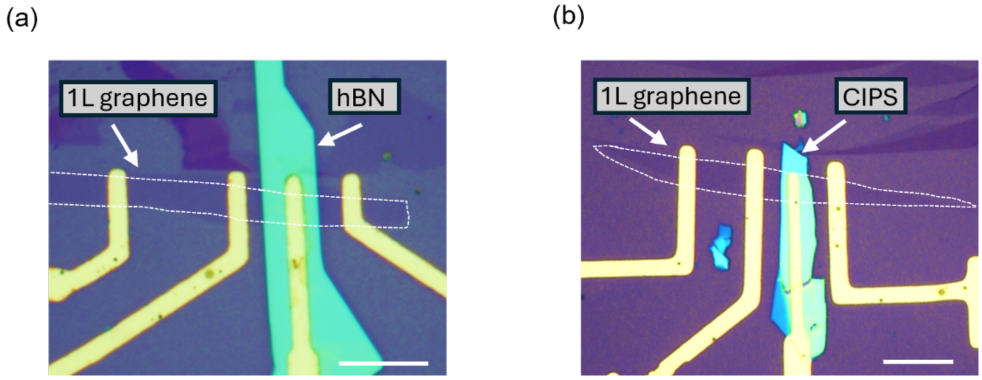

Figure S4. (a) Optical microscope image of two graphene field effect transistor (FET) devices fabricated using In/Au metal electrodes on the same monolayer graphene flake. One of the FETs is covered with a 49 nm thick hBN flake. (b) Optical microscope image of two graphene FET devices fabricated using In/Au metal electrodes on the same monolayer graphene flake. One of the FETs is covered with a 45 nm thick CIPS flake. Scale bar is 10 µm.

Section 5: Output characteristics for SiO_2 gated graphene FET device

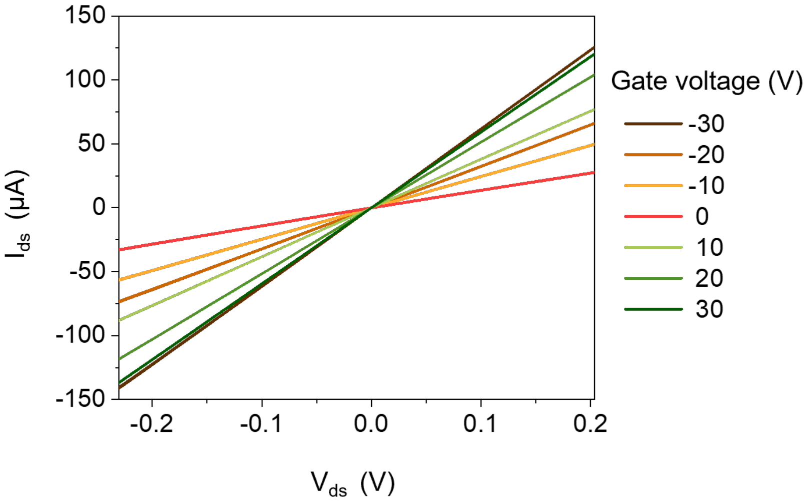

Figure S5. I_{ds}–V_{ds} (drain current–drain voltage) characteristics of SiO_2 gated graphene FET device at different back gate voltages. The measurements were performed in vacuum and at room temperature.

Section 6: Mechanism of Cu^+ ion movement in CIPS

The spatial instability of monovalent Cu^+ ion leads to out-of-plane ferroelectricity in CIPS.[1,2] **Figure S6** shows the position of the Cu^+ ion within the sulphur octahedra when CIPS is polarized in 'up' and 'down' directions. When the polarization points downwards, the Cu^+ ion is in proximity to the graphene layer resulting in localized electron doping. The displacement of the Cu^+ cation is dependent on temperature.[3] At higher temperatures, the Cu^+ ion penetrates to the vdW gap and contributes to ionic conductivity.[4,5] As a result, the electron doping is delocalized causing the second resistivity peak to move towards the primary Dirac peak as observed in **Figure 3b,c** in the main manuscript.

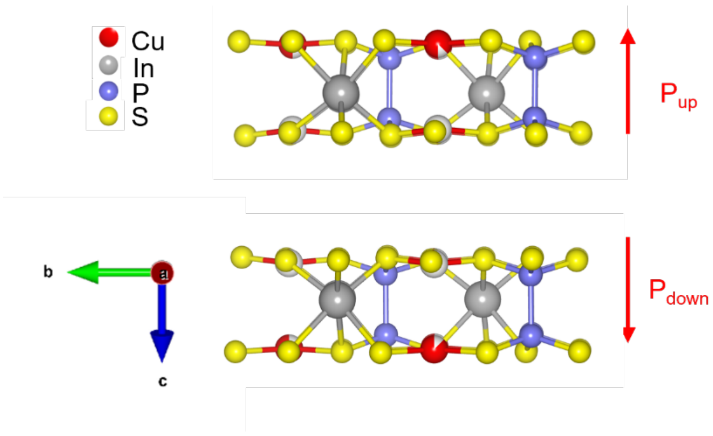

Figure S6. Movement of Cu^+ ion within sulphur octahedra for upward and downward polarization directions.

Section 7: Temperature dependent transfer characteristics of bare graphene FET

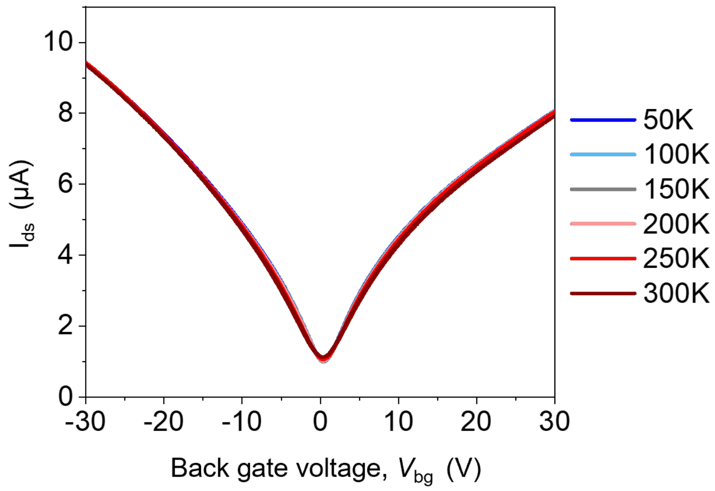

Figure S7. I_{ds} $-V_{gs}$ (drain current–gate voltage) characteristics of SiO_2 gated graphene FET device at different temperature. The measurements were performed in vacuum.

REFERENCES

1. V. Maisonneuve, M. Evain, C. Payen, V. B. Cajipe, P. Molinic, Room-temperature crystal structure of the layered phase. J. Alloys Compd. (1995).
2. V. Maisonneuve, V. B. Cajipe, A. Simon, R. Von Der Muhll, J. Ravez, Ferrielectric ordering in lamellar $CuInP_2S_6$. Physical Review B 56, 17 (1997).
3. S. M. Neumayer, L. Tao, L. Tao, A. O'hara, J. Brehm, M. Si, M. Si, P. Y. Liao, P. Y. Liao, T. Feng, T. Feng, S. V. Kalinin, P. D. Ye, P. D. Ye, S. T. Pantelides, P. Maksymovych, N. Balke, Alignment of polarization against an electric field in van der waals ferroelectrics. Phys Rev Appl. 13 (2020).
4. Z. Zhou, S. Wang, Z. Zhou, Y. Hu, Q. Li, J. Xue, Z. Feng, Q. Yan, Z. Luo, Y. Weng, R. Tang, X. Su, F. Zheng, K. Okamoto, H. Funakubo, L. Kang, L. Fang, L. You, Unconventional polarization fatigue in van der waals layered ferroelectric ionic conductor $CuInP_2S_6$. Nat Commun. 14 (2023).
5. D. D. Xu, R. R. Ma, Y. F. Zhao, Z. Guan, Q. L. Zhong, R. Huang, P. H. Xiang, N. Zhong, C. G. Duan, Unconventional out-of-plane domain inversion *via* in-plane ionic migration in a van der Waals ferroelectric J Mater Chem C Mater. 8, 6966–6971 (2020).
